# Hip Dislocation With Femoral Short Neck in Spondyloepiphyseal Dysplasia Treated by Open Reduction and Valgus Osteotomy: A Case Report

**DOI:** 10.7759/cureus.103858

**Published:** 2026-02-18

**Authors:** Akitoshi Sakuma, Jun Kakizaki, Yasuhiro Oikawa

**Affiliations:** 1 Division of Orthopedic Surgery, Chiba Children’s Hospital, Chiba, JPN

**Keywords:** case report, developmental dislocation of the hip, femoral short neck, femoral valgus osteotomy, spondyloepiphyseal dysplasia congenita

## Abstract

Spondyloepiphyseal dysplasia congenita (SEDC) often involves hip deformity and dislocation. We present a case of an eight-year-old boy with bilateral femoral head deformity and hip dislocation treated by open reduction and femoral valgus osteotomy. Surgery was performed via an anterior approach. After removal of obstructive factors, reduction was only maintained at 30° adduction, necessitating valgus osteotomy to stabilize the hip. Postoperative fixation was achieved with hip spica casting. This case suggests that femoral valgus osteotomy may be essential to maintain reduction in SEDC-associated hip dislocation.

## Introduction

Spondyloepiphyseal dysplasia congenita (SEDC) is a rare skeletal disorder caused by mutations in the *COL2A1* gene, leading to abnormal synthesis of type II collagen. This defect results in a spectrum of clinical manifestations, including short-trunk dwarfism, platyspondyly, and epiphyseal dysplasia [1,2]. Among these, hip joint abnormalities such as coxa vara and acetabular dysplasia often lead to gait disturbances and secondary osteoarthritis [3]. Consequently, surgical interventions, including valgus osteotomy of the femur [4] and total hip arthroplasty, may be required [5]. Separately from SEDC, in cases of hip dislocation, open reduction (OR) is frequently performed in combination with femoral varus osteotomy [6,7]. In this report, we present a case of bilateral hip dislocation secondary to hip deformities associated with SEDC, namely, short femoral neck, high-riding greater trochanter, and acetabular dysplasia. The patient underwent OR combined with femoral valgus osteotomy, resulting in favorable clinical outcomes.

## Case presentation

**Figure 1:**
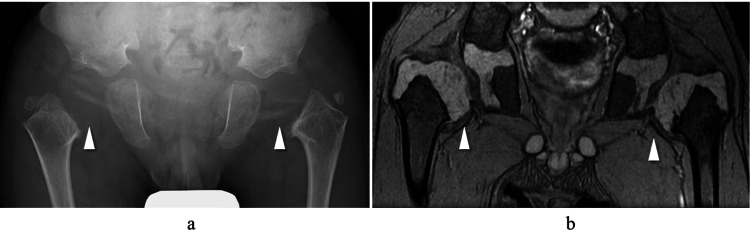
Plain radiograph and MRI image at age 4. (a) Plain radiograph: The ossification center is not visible (white arrowheads), and lateral displacement of the proximal femur is observed. (b) MRI T2*-weighted image: The femoral neck is shortened, and the femoral head is subluxated (white arrowheads).

An eight-year-old boy was diagnosed with SEDC based on clinical features and imaging findings. Since the onset of walking at age 2 years, a waddling gait had been observed, and plain radiographs suggested bilateral coxa vara. At the age of 4, a feeling of hip subluxation and spontaneous reduction while walking was noted in both hips. Plain radiographs revealed lateral displacement of both femoral heads, and magnetic resonance imaging (MRI) demonstrated shortening of the femoral necks and subluxation of the hip joints (Figure 1).

**Figure 2:**
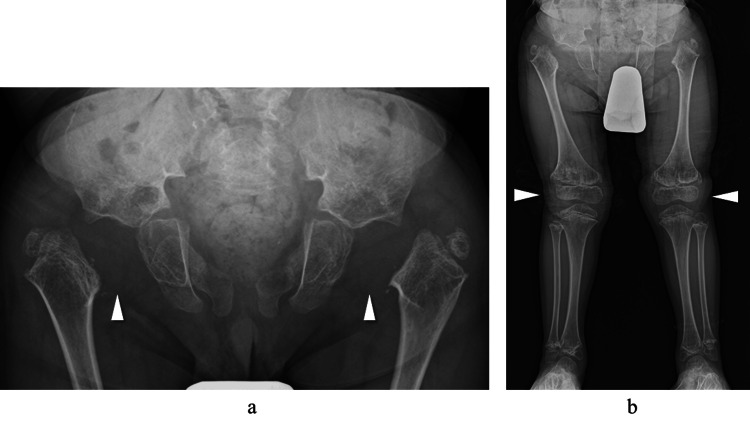
Plain radiographs at age 8. (a) Anteroposterior view of the hips: The femoral head is not visible, and lateral displacement of the proximal femur has progressed (white arrowheads). (b) Standing full-length view of the lower limbs: Bilateral genu valgum is observed (white arrowheads).

At that time, we proposed surgical intervention for hip subluxation; however, as the patient did not experience pain, neither the patient nor their family wished to proceed with surgery. Furthermore, marked thickening of the acetabular floor and a small acetabulum were considered unfavorable for achieving stability through OR, and thus observation was continued. By the age of 8, the patient exhibited a pronounced anterior pelvic tilt during gait. Plain radiographs revealed progression of proximal femoral lateralization and the presence of genu valgum (Figure 2).

**Figure 3:**
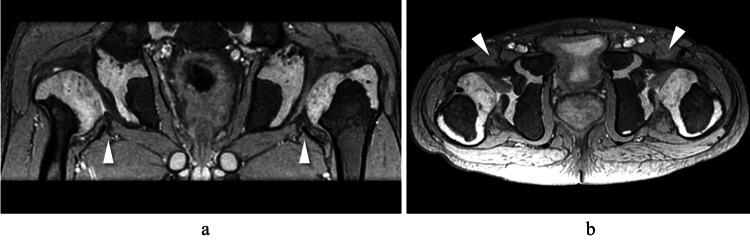
MRI T2*-weighted images at age 8. (a) Coronal view: Progressive lateral displacement of the femoral head is observed (white arrowheads). (b) Axial view: Hypertrophied ligamentum teres and limbus are identified (white arrowheads).

MRI demonstrated further subluxation of the femoral heads, along with hypertrophy of the ligamentum teres and limbus (Figure 3), which were considered contributing factors to the subluxation.

At this point, the patient and their family requested surgical intervention, with the primary aim of improving gait function. Consequently, bilateral OR and guided growth surgery of the distal medial femur were performed.

Surgical technique

**Figure 4:**
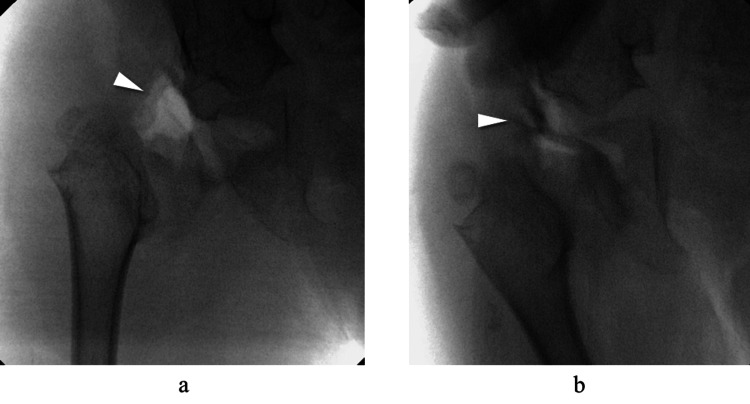
Intraoperative fluoroscopic images with intra-articular contrast. (a) Neutral position: The femoral head is dislocated (white arrowhead). (b) 30° adduction position: The femoral head is successfully reduced into the acetabulum (white arrowhead).

First, surgery was performed on the right hip. Under general anesthesia, the patient was positioned with a towel placed beneath the lower back to elevate the pelvis. The surgical approach utilized a modified Smith-Peterson technique [8] through a 5 cm incision along the inguinal crease. The interval between the tensor fasciae latae and sartorius muscles was developed, and the rectus femoris was proximally detached and later repaired prior to wound closure. Portions of the gluteus medius and minimus were elevated subperiosteally from the iliac bone to expose the hip joint capsule. Upon capsulotomy and intra-articular inspection, hypertrophy of the ligamentum teres and protrusion of the limbus were identified as obstacles to reduction. Therefore, the ligamentum teres was excised, and the limbus was incised radially to create sufficient space for femoral head reduction into the acetabulum. At this point, reduction was achievable; however, it could only be maintained with the hip positioned in approximately 30° of adduction (Figure 4).

**Figure 5:**
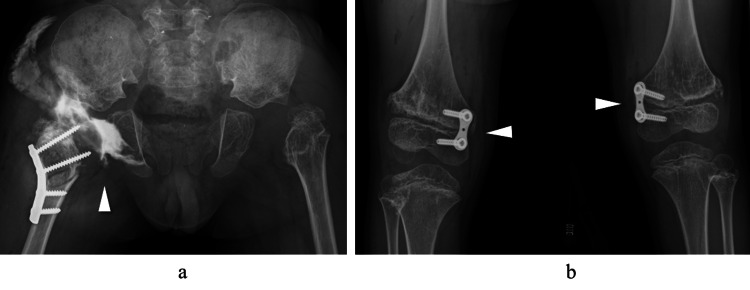
Postoperative plain radiographs. (a) Anteroposterior view of the hip: The femoral head remains reduced even in slight abduction of the hip (white arrowhead). (b) Anteroposterior view of the knee: Guided growth was performed using a tension band plate on the distal medial femur (white arrowheads).

To preserve the reduction position, a femoral valgus osteotomy of approximately 30° was added. As a plate designed specifically for valgus osteotomy was not available, a dynamic compression plate (Depuy-Synthes, Warsaw, IN, USA) was bent to 40° and used for fixation (Figure 5).

Additionally, guided growth surgery was performed for bilateral genu valgum using a tension band plate (Eight-Plate®, Orthofix, Verona, Italy), which was applied to the distal medial femur. Postoperatively, the hip was immobilized in a mildly abducted position using a hip spica cast to maintain the reduction. The operative time was 5 hours and 5 minutes, with a total anesthesia duration of 8 hours and 3 minutes. Intraoperative blood loss was 73 ml. After six weeks of hip immobilization with a hip spica cast, gait training was initiated.

After confirming bone union at the osteotomy site, the same surgical procedure - OR and femoral valgus osteotomy - was performed on the left side six months after the initial operation. For this procedure, a Pediatric LCP™ Plate designed for valgus osteotomy (Depuy-Synthes, Warsaw, IN, USA) was utilized. The operative time was 3 hours and 19 minutes, the total anesthesia duration was 5 hours and 38 minutes, and the intraoperative blood loss was 119 ml.

**Figure 6:**
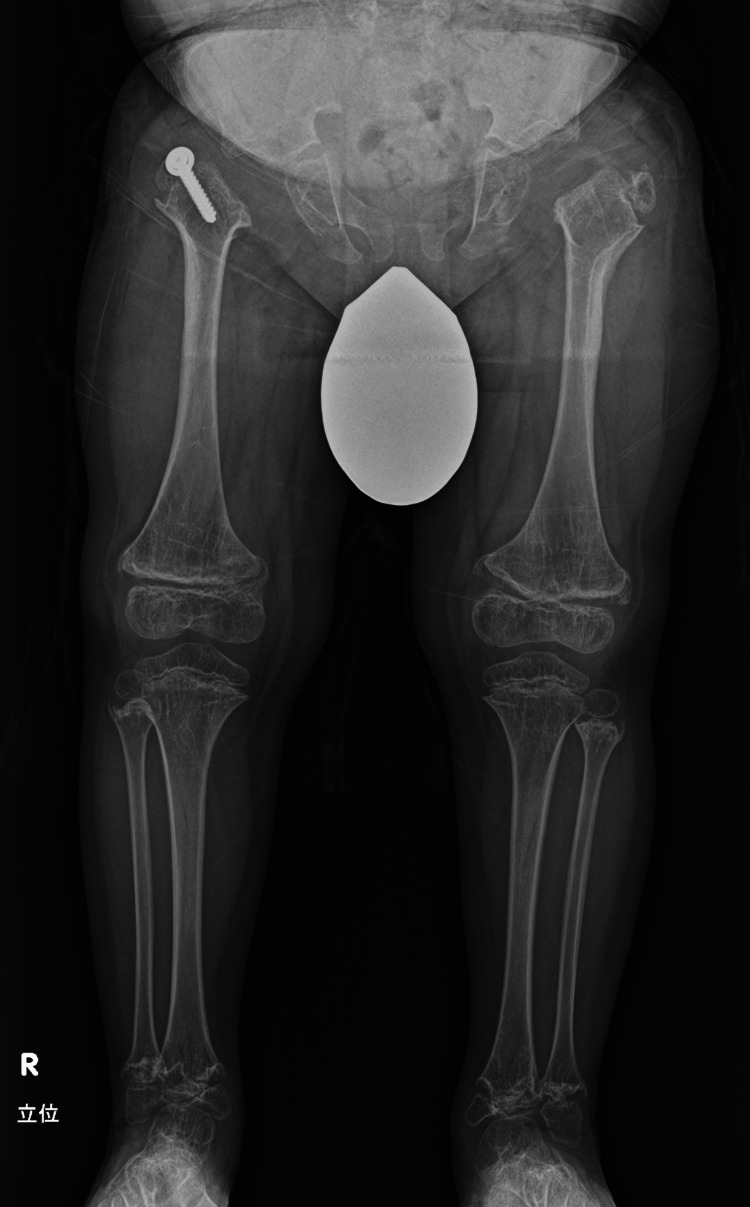
A standing full-length view of the lower limbs at 3 years postoperatively. Lower limb alignment is satisfactory, and no recurrence of hip dislocation is observed. A greater trochanteric advancement procedure was performed on the right side.

One year and eight months postoperatively, improvement in lower limb alignment and bone union was confirmed, and the proximal and distal femoral plates were removed. Additionally, on the right side, a greater trochanteric advancement procedure was performed using a screw to address a high-riding greater trochanter. At three years postoperatively, satisfactory lower limb alignment was maintained, and no recurrence of hip dislocation was observed (Figure 6).

The patient's gait had improved, and the click sign of the hip had resolved.

## Discussion

Hip deformities associated with SEDC typically include coxa vara and acetabular dysplasia [1,2]. However, to the best of our knowledge, this is the first reported case presenting with femoral neck shortening and hip dislocation.

In general, surgical treatment for hip dislocation after starting of walk involves OR combined with pelvic osteotomy, femoral varus osteotomy, or femoral shortening [6,7]. Nevertheless, we were unable to identify any previous reports describing the use of OR in combination with femoral valgus osteotomy, as performed in the present case.

In this case, anatomical interference between the greater trochanter and the pelvis in the neutral hip position prevented proper reduction. However, adduction of the hip allowed satisfactory reduction, prompting the decision to perform a femoral valgus osteotomy.

For hip dysplasia and osteoarthritis associated with similar deformities, including femoral neck shortening and high-riding greater trochanter, a previous report described the use of combined pelvic osteotomy and femoral valgus osteotomy [9].

In contrast, in this case, femoral valgus osteotomy and OR were sufficient to maintain the reduced position of the hip. Unlike their report, obstructive factors at the acetabular floor - hypertrophied ligamentum teres and limbus - were removed during OR. As a result, adequate femoral head coverage was achieved in the reduced position, and pelvic osteotomy was not required to maintain the reduction.

As the postoperative follow-up period in this case remains relatively short, careful long-term monitoring is necessary to watch for potential recurrence of dislocation or the development of osteoarthritis.

## Conclusions

In this case of hip deformity associated with SEDC, OR and femoral valgus osteotomy were performed simultaneously. OR is typically combined with a varus osteotomy, but in this case, a valgus osteotomy was performed to stabilize the hip joint, effectively addressing the underlying dysplasia. Although such cases are extremely rare, femoral valgus osteotomy in addition to OR may be effective in managing hip dislocation associated with hip deformities in SEDC.
